# Modified Spinous Process–Splitting Approach for Thoracolumbar Burst Fractures With Neurological Deficits: Technical Description and Preliminary Clinical Outcomes

**DOI:** 10.1111/os.70316

**Published:** 2026-04-19

**Authors:** Kaixuan Chen, Yizhong Ma, Zihui Yang, Hongfeng Ruan, Guanyi Liu

**Affiliations:** ^1^ Health Science Center, Ningbo University Ningbo China; ^2^ The First Affiliated Hospital, Zhejiang Chinese Medical University Hangzhou China; ^3^ Department of Spine Surgery Center, Ningbo Ningbo China; ^4^ Ningbo Clinical Research Center for Orthopedics, Sports Medicine & Rehabilitation Ningbo China

**Keywords:** muscle‐sparing technique, neurological deficit, posterior decompression, spinous process–splitting approach, thoracolumbar burst fracture, Wiltse approach

## Abstract

**Background:**

Thoracolumbar burst fractures with neurological deficits require decompression and stabilization. The spinous process‐splitting approach (SPSA) achieves neural decompression while preserving the posterior osteoligamentous complex and minimizing paraspinal muscle injury.

**Objective:**

Although various posterior surgical techniques are available for thoracolumbar burst fractures with neurological deficits, achieving adequate neural decompression while maximally preserving the posterior osteoligamentous complex remains technically challenging; a standardized approach that balances sufficient decompression with posterior structural preservation is still lacking. Therefore, we aimed to describe a modified SPSA combining the Wiltse interval with spinous process splitting for decompression, and to preliminarily evaluate its feasibility, safety, and early clinical and radiographic outcomes.

**Methods:**

We retrospectively reviewed seven consecutive male patients who underwent posterior fixation, decompression, and fusion via modified SPSA between January 2020 and December 2024. Outcomes included American Spinal Injury Association (ASIA) grade, Visual Analog Scale (VAS) for pain, Gardner angle, anterior vertebral height ratio, and spinal canal encroachment ratio, assessed preoperatively, at 1 week postoperatively, and at 14 ± 2 months. Repeated‐measures ANOVA was performed (α = 0.05).

**Results:**

All procedures were completed successfully with mean operative time of 172.1 ± 95.7 min. All patients improved by at least one ASIA grade postoperatively, with no neurological deterioration. VAS scores decreased from 6.86 ± 0.90 preoperatively to 3.29 ± 0.49 at 1 week, and to 1.14 ± 0.38 at final follow‐up (*F* = 350.00, *p* < 0.01). Gardner angle improved from 19.63° ± 8.92° to 4.39° ± 4.08° and remained stable at 4.69° ± 4.27° (*F* = 54.65, *p* < 0.01). Anterior vertebral height ratio increased from 51.82% ± 1.20% to 91.96% ± 7.93% and was maintained at 91.30% ± 8.13% (*F* = 89.41, *p* < 0.01). Spinal canal encroachment decreased from 62.32% ± 14.90% to 16.42% ± 10.78% postoperatively and remained stable at 16.89% ± 11.54% (*F* = 48.49, *p* < 0.01). All fractures achieved radiographic union without loss of correction.

**Conclusions:**

Modified SPSA combining the Wiltse interval and midline spinous process splitting appears feasible for achieving decompression, reduction, and fixation while preserving posterior structures in selected patients with thoracolumbar burst fractures and neurological deficits. Larger prospective comparative studies are required to confirm long‐term efficacy and define optimal patient selection criteria.

## Graphical abstract

A modified spinous process–splitting approach provides effective midline decompression with preservation of posterior structures, resulting in marked neurological improvement, reduced pain, and substantial correction of canal encroachment in thoracolumbar burst fractures.

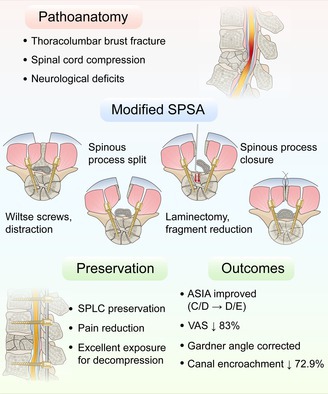

## 1 Introduction

Thoracolumbar burst fractures represent a severe spinal injury, typically resulting from high‐energy trauma such as motor vehicle accidents or falls [1]. Despite extensive research and guidelines, considerable variation persists between recommended and actual treatment strategies for thoracolumbar burst fractures [2]. The role of surgery in treating thoracolumbar burst fractures without neurological deficits remains controversial. Studies have shown that surgery does not consistently provide superior pain relief or functional recovery compared with conservative management [3]. However, surgical treatment may provide better radiographic correction and earlier mobilization, whereas conservative management may lead to progressive deformity in some patients [4]. Therefore, clinical decision‐making depends on injury classification systems and patient‐specific factors.

For surgical candidates, evidence remains insufficient to establish the long‐term superiority of any single approach, as each has distinct limitations. The anterior approach provides direct access to the anterior column and allows removal of retropulsed fragments but requires transthoracic or retroperitoneal exposure, increasing risks of visceral and vascular injury [5]. The posterior midline approach allows straightforward reduction and fixation but requires extensive muscle dissection and disruption of the posterior ligamentous complex, leading to increased blood loss, postoperative pain, multifidus atrophy, and chronic low back pain [6]. Combined approaches allow reconstruction of both columns but involve longer operative time, greater blood loss, and higher perioperative morbidity [7]. Given these limitations, posterior techniques that minimize tissue injury while providing adequate neural decompression and stabilization warrant further investigation.

The Wiltse interval, a muscle‐sparing posterior approach, avoids midline osseoligamentous structures and preserves the posterior ligamentous complex while permitting pedicle screw placement and fracture reduction [8]. Compared with the midline open approach, the Wiltse interval achieves comparable stability with substantially reduced soft‐tissue disruption [9]. Nonetheless, this technique limits direct midline exposure, restricting the ability to effectively address central or contralateral retropulsed fragments. Contralateral decompression through a unilateral approach often requires manipulation at steep angles, increasing the risk of dural tears [10]. The spinous process–splitting approach (SPSA) addresses these limitations. SPSA involves midline splitting of the spinous process, providing direct access for decompression. Simultaneously, this approach preserves multifidus attachments and minimizes muscle injury and postoperative pain [11, 12]. SPSA has been widely adopted for posterior decompression in lumbar spinal stenosis [13]. Randomized controlled trials have shown that SPSA provides better early pain control and functional recovery compared with conventional laminectomy, with comparable long‐term outcomes [14].

We therefore developed a combined surgical strategy integrating the Wiltse paraspinal intermuscular interval with SPSA for thoracolumbar burst fractures with neurological deficits. The Wiltse approach facilitates muscle‐sparing fixation and reduction, while SPSA provides midline decompression without disrupting the posterior tension band. We hypothesized that this modified approach would achieve adequate decompression and stabilization while preserving posterior structures. Therefore, the purposes of this study were: (i) to describe the surgical technique of the modified SPSA in detail and explore the technical feasibility and perioperative safety; (ii) to provide a comprehensive assessment of early clinical and radiographic outcomes; and (iii) to offer spine surgeons a reproducible, muscle‐sparing alternative surgical approach for managing thoracolumbar burst fractures with neurological deficits.

## 2 Patients and Methods

### 2.1 Study Design and Participants

We retrospectively reviewed seven consecutive male patients with thoracolumbar burst fractures with neurological deficits who underwent posterior fixation, decompression, and fusion via modified SPSA between January 2020 and December 2024. The study protocol was approved by the Ethical Review Board of our institution (approval no. 202335K). No female patients met the inclusion criteria during the study period; therefore, sex‐specific exclusion criteria were not established. Inclusion criteria were: (1) traumatic thoracolumbar burst fractures confirmed by radiography, computed tomography (CT), and magnetic resonance imaging (MRI); (2) neurological deficits (American Spinal Injury Association (ASIA) grade C or D); (3) complete preoperative and postoperative clinical and radiologic data; and (4) treatment with the combined SPSA technique. Exclusion criteria were: pathological or osteoporotic fractures, multiple injuries with hemodynamic instability, history of prior thoracolumbar trauma or spinal surgery, and incomplete clinical or radiological data, including loss to follow‐up.

All patients presented with severe low back pain accompanied by unilateral or bilateral lower limb numbness and weakness. The mechanism of injury in all cases was a fall from height. Fracture distribution included three single‐level fractures (L3, *n* = 2; L1, *n* = 1) and four multi‐level fractures (L1–L2, L2–L3, T12–L1, and T12–L3; *n* = 1 each). Using the AO Spine classification, two fractures were A3 with N2, and five were classified A3 with N3 [15]. Preoperative ASIA grades were C (*n* = 5) and D (*n* = 2). Preoperative imaging showed vertebral fractures, kyphotic deformity, and retropulsed bone fragments encroaching into the spinal canal, resulting in compression of the dural sac and/or nerve roots (Table 1).

**TABLE 1:** Baseline patient characteristics and perioperative outcomes.

ID	Gender	Age	Fracture level	Fixed level	AOSpine type	ASIA grade			Complications	Operative time (min)	Estimated blood loss (mL)	Hospital stay (days)
						Pre‐operative	1 week post‐op	14 ± 2 months post‐op				
1	M	48	L1, L2	T11‐L4	A3, N3	C	D	D	None	192	800	12
2	M	51	L3	T12‐L5	A3, N3	D	E	E	None	120	600	25
3	M	53	L2, L3	L1‐L5	A3, N3	C	D	D	None	200	700	13
4	M	35	L3	L1‐L5	A3, N3	C	D	D	None	360	300	21
5	M	60	L1	T11‐L1	A3, N2	C	D	D	None	60	100	13
6	M	49	T12, L1	T11‐L3	A3, N3	C	D	D	None	155	900	11
7	M	54	T12, L3	T11‐L4	A3, N2	D	E	E	None	118	600	11

Visual Analog Scale (VAS) scores for low back pain were recorded: preoperatively, at 1 week, and at 14 ± 2 months postoperatively. Radiographic parameters were measured on CT images. On sagittal CT images, we measured the Gardner angle (the angle between the superior endplate of the cranial vertebra and the inferior endplate of the fractured vertebra) and anterior vertebral height. The anterior height compression ratio was calculated as: (mean anterior height of adjacent vertebrae − anterior height of fractured vertebra)/mean anterior height of adjacent vertebrae × 100%. On axial CT images, the spinal canal encroachment ratio, defined as the proportion of canal area occupied by retropulsed fragments was measured, providing a comprehensive evaluation of fracture‐related morphological changes [16]. All CT examinations were performed using a Siemens SOMATOM Definition AS scanner (Siemens Healthcare, Erlangen, Germany) with the following parameters: 120 kV, 200–250 mA, slice thickness 1.0 mm, and reconstruction interval 1.0 mm. Radiographic measurements, including Gardner angle, anterior vertebral height, and spinal canal encroachment ratio, were performed on sagittal and axial CT images using the institutional Picture Archiving and Communication System (PACS) workstation. All measurements were independently conducted by two spine surgeons with more than 10 years of experience, who were blinded to the clinical and surgical data. To assess measurement reliability, intra‐ and inter‐observer intraclass correlation coefficients (ICC) were calculated. The results demonstrated excellent reliability for all parameters (ICC > 0.90), indicating high measurement accuracy and consistency.

Statistical analyses were performed using SPSS version 25.0 (IBM Corp., Armonk, NY, USA). Continuous variables were expressed as mean ± SD. Data normality was assessed using the Shapiro–Wilk test, and Mauchly's test of sphericity was applied for repeated‐measures comparisons. When the assumption of sphericity was violated (*p* < 0.05), the Greenhouse–Geisser correction was used. For normally distributed data, a one‐way repeated‐measures ANOVA was performed to compare preoperative, 1‐week postoperative, and final follow‐up outcomes, followed by Bonferroni‐adjusted post hoc tests. Effect sizes (partial *η* ^2^) and 95% confidence intervals (CIs) were calculated for all major outcome measures (VAS, Gardner angle, anterior height compression ratio, and spinal canal encroachment ratio). Statistical significance was defined as *p* < 0.05.

### 2.2 Surgical Technique

Following induction of general anesthesia with endotracheal intubation, patients were positioned prone with the lumbar spine hyperextended using bolsters to maintain lordotic curvature and facilitate indirect fracture reduction through ligamentotaxis. A posterior midline skin incision was made, followed by bilateral subcutaneous dissection to identify and expose the Wiltse intermuscular interval between the multifidus and longissimus muscles. Pedicle screw entry points were then identified and prepared. The selection of fixation segments was determined according to the fracture type, degree of comminution, and segmental stability. In most cases, fixation was performed at two levels above and two levels below the fractured vertebra; for cases with severe comminution or marked endplate collapse, the fixation range was appropriately extended. Accurate pedicle screw positioning was confirmed intraoperatively using C‐arm fluoroscopy.

Subsequently, rods were connected to the pedicle screws, and fracture reduction was achieved through distraction before locking the rod‐screw system in place. To expose the decompression area, the tips of the spinous processes were longitudinally split using an osteotome, and the split processes were carefully detached at the base along with the interspinous ligament. These structures were retracted laterally, clearly exposing the laminae. A complete laminectomy was performed to fully visualize the spinal canal and dura mater, enabling thorough exploration. Bone fragments intruding into the spinal canal and posteriorly displaced vertebral body fragments were carefully reduced. Dural tears by fracture fragments were repaired as needed. Autologous bone fragments obtained during decompression were used for posterolateral fusion in all cases. For patients with severe intervertebral disc destruction or significant segmental instability identified on preoperative imaging or intraoperatively, posterior lumbar interbody fusion was additionally performed, and the intervertebral space was filled with a mixture of autologous and allogeneic bone granules. Following decompression and fusion, all patients underwent computed tomography at final follow‐up, which confirmed solid bony fusion, with a fusion rate of 100%.

**FIGURE 1:**
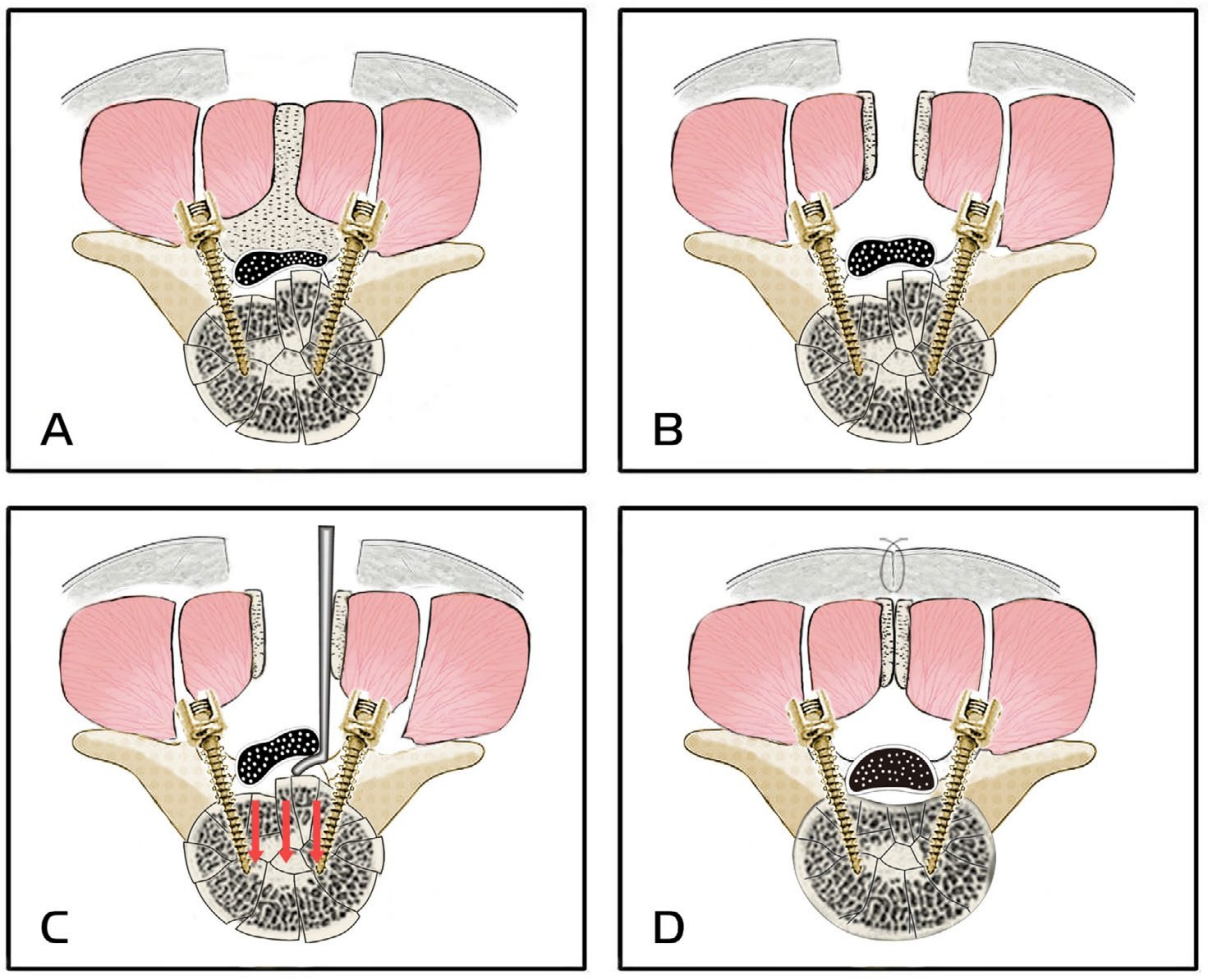
Schematic diagram illustrating the modified SPSA. (A) Pedicle screws were bilaterally inserted through the Wiltse interval, and the fracture was reduced by distraction. (B) The spinous process was exposed and longitudinally split. (C) Laminectomy was performed, and fracture fragments encroaching on the spinal canal were reduced through the posterior approach. (D) The spinous process was sutured, and the incision was closed.

After decompression, the split spinous processes were repositioned and securely sutured. A negative‐pressure drainage tube was placed, and the incision was meticulously closed in layers, ensuring watertight closure of both fascial and cutaneous tissues (Figure 1).

### 2.3 Postoperative Management

Prophylactic antibiotics were routinely administered for 24–48 h postoperatively to prevent infection. Patients were advised to maintain bed rest for ~2 days following surgery to ensure initial stabilization and adequate pain management. Once pain had sufficiently subsided and patients demonstrated adequate recovery, gradual mobilization commenced under the support of a lumbar brace, starting with gentle activities such as sitting and standing. Continuous brace usage was required until radiographic evidence confirmed fracture healing, typically at ~3 months, to ensure proper stabilization during the recovery period. All patients completed follow‐up, with a follow‐up rate of 100%. Follow‐up evaluations were conducted through outpatient visits, telephone interviews, and imaging examinations. The minimum and maximum follow‐up durations were 12 and 16 months, respectively (mean, 14 ± 2 months).

### 2.4 Clinical Presentation

**FIGURE 2:**
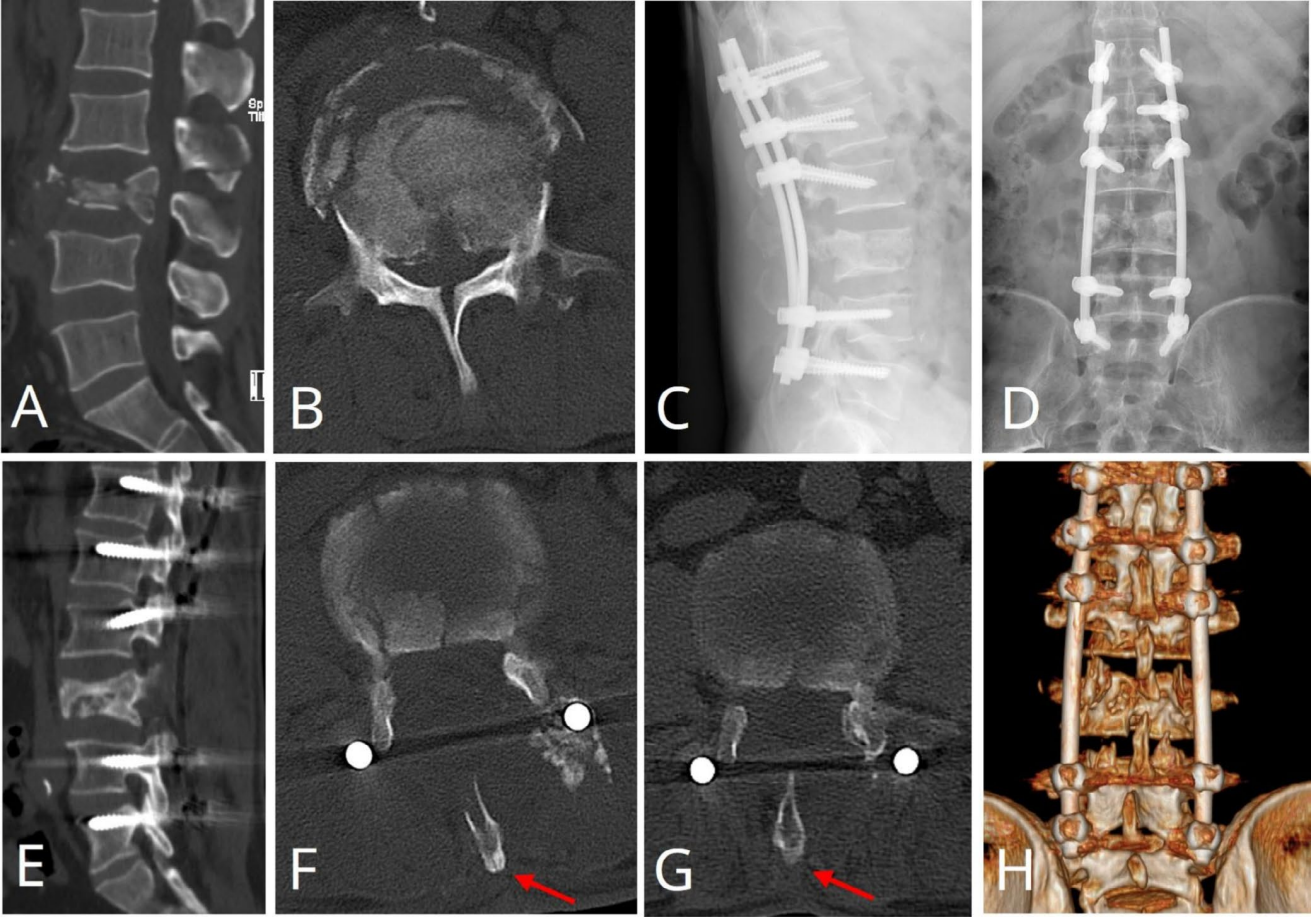
Preoperative and postoperative imaging of Case 1. Preoperative CT scans showing an L3 vertebral burst fracture with partial retropulsion of bone fragments into the spinal canal, causing spinal cord compression (A, B). Anteroposterior and lateral radiographs at 12 months postoperatively demonstrating proper placement of the pedicle screw‐rod fixation system and fracture union (C, D). Sagittal and axial CT images at 12 months postoperatively reveal adequate decompression of the spinal canal, fracture healing, and union of the split spinous process (red arrows) (E–G). A reconstructed CT image illustrates osseous union at the L3 fracture (H).

Case 1 A 51‐year‐old male presented with an L3 vertebral burst fracture complicated by incomplete paralysis (ASIA grade D) following a fall from height. Preoperatively, the patient had a low back pain VAS score of 8, a spinal canal encroachment ratio of 61.54%, and an anterior vertebral height ratio of 41.61%. At 12 months postoperatively, after treatment using the modified SPSA, neurological function improved to ASIA grade E, the VAS score decreased to 1, the spinal canal encroachment ratio decreased to 13.59%, and the anterior vertebral height ratio increased to 86.96% (Figure 2).

**FIGURE 3:**
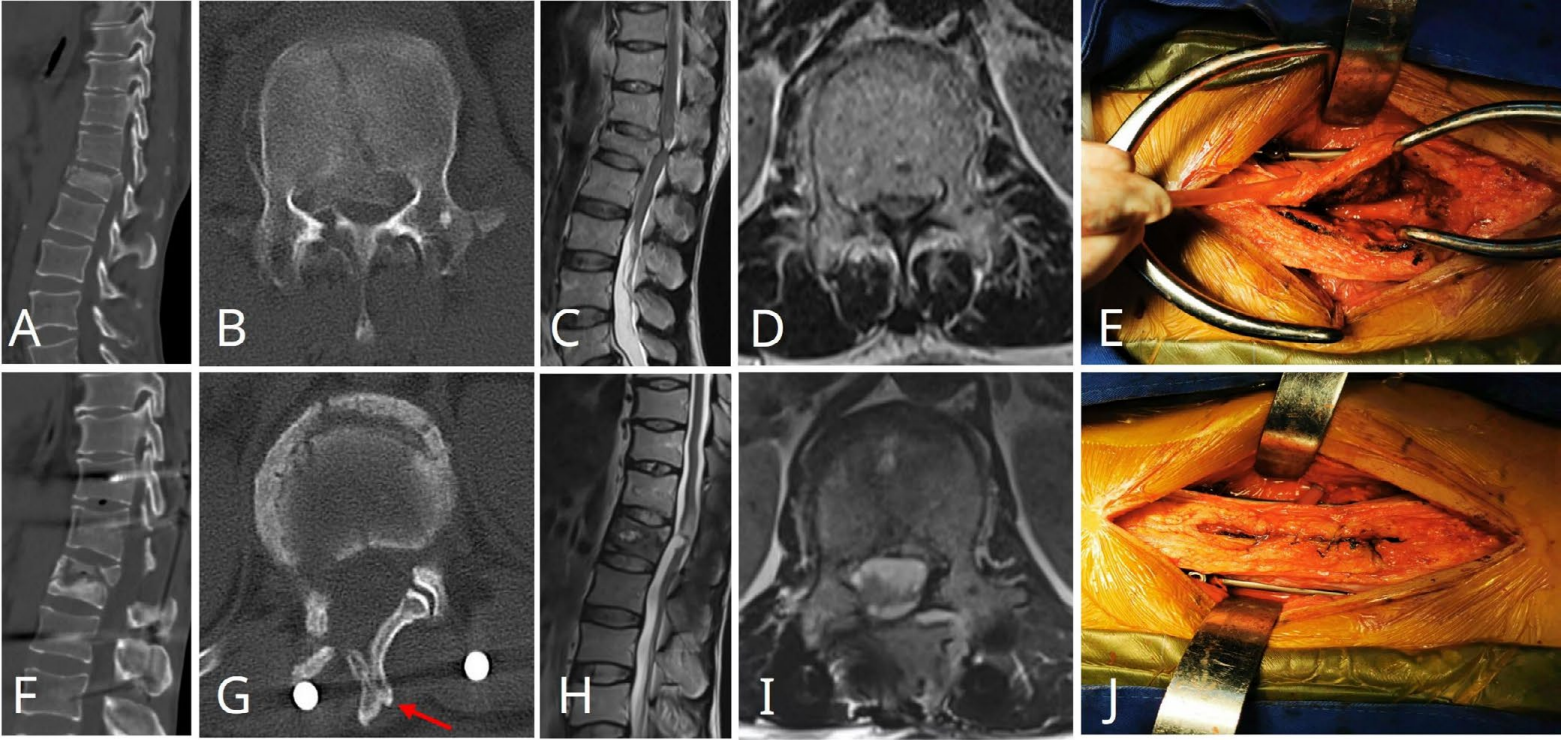
Preoperative and postoperative imaging of Case 2. Preoperative CT (A, B) and MRI (C, D) demonstrating an L1 burst fracture with retropulsion of bone fragments into the spinal canal, causing significant spinal cord compression. Postoperative CT (F, G) and MRI (H, I) images confirming fracture healing and union of the split spinous process (red arrow). Intraoperative photograph illustrating fracture fixation and reduction through the Wiltse interval, followed by decompression and realignment via SPSA (E). After decompression, the split spinous process was meticulously sutured (J).

Case 2 A 49‐year‐old male sustained an L1 vertebral burst fracture complicated by conus medullaris syndrome due to a fall from height. Preoperative assessment indicated ASIA grade C, VAS score of 8 for low back pain, spinal canal encroachment ratio of 73.52%, anterior vertebral height ratio of 42.31%, and Gardner angle of 30.59°. After fixation and decompression surgery using the combined Wiltse interval and SPSA technique, neurological function improved to ASIA grade D at final follow‐up, VAS score decreased to 2, spinal canal encroachment ratio decreased to 10.27%, anterior vertebral height ratio recovered to 98.08%, and Gardner angle improved to 9.12°. This case demonstrates that the described surgical technique effectively corrects kyphotic deformity and achieves adequate neural decompression in thoracolumbar burst fractures with neurological impairment (Figure 3).

## 3 Results

### 3.1 Perioperative Outcomes

All seven patients successfully underwent fixation, decompression, and fusion using the modified SPSA, with a mean operative time of 172.1 ± 95.7 min (range, 60–360 min). Approximately 90 ± 25 min were spent on Wiltse paraspinal exposure and fixation, 45 ± 10 min on decompression using the modified SPSA, and the remaining time on irrigation, bone grafting, and wound closure. The mean estimated intraoperative blood loss was 571 ± 260 mL (range, 100–900 mL), and the mean hospital stay was 15.1 ± 5.6 days (range, 11–25 days).

### 3.2 Neurological Outcomes and Complications

At final follow‐up, all patients demonstrated at least a one‐grade improvement in ASIA classification compared with their preoperative status. Five patients improved from ASIA grade C to D, and two patients improved from ASIA grade D to E. No patient experienced neurological deterioration. Four patients (57.1%) were found to have dural tears caused by fracture fragments; all were recognized and repaired intraoperatively with watertight dural closure. One patient developed postoperative cerebrospinal fluid leakage, which resolved completely with conservative management (bed rest and pressure dressing) without prolonging hospitalization or causing secondary infection. No wound infections, implant‐related complications, or other major complications were observed during follow‐up (Table 1). At the final follow‐up, no recurrent neurological deterioration or kyphotic deformity progression was observed (Table 2).

**TABLE 2:** Clinical and radiographic outcomes.

Evaluation time	VAS for back pain (mean ± SD; 95% CI)	% Change from baseline	Gardner angle (mean ± SD; 95% CI)	% Change from baseline	Anterior vertebral height ratio (mean ± SD; 95% CI)	% Change from baseline	Spinal canal encroachment ratio (mean ± SD, 95% CI)	% Change from baseline
Pre‐operative	6.86 ± 0.90 (6.28–7.44)	—	19.63 ± 8.92 (13.46–25.80)	—	51.82 ± 1.2 (50.89–52.75)	—	62.32 ± 14.90 (51.84–72.80)	—
1 week post‐op	3.29 ± 0.49 (2.95–3.63)	−52.1%	4.39 ± 4.08 (1.35–7.43)	−77.6%	91.96 ± 7.93 (86.25–97.67)	+77.5%	16.42 ± 10.78 (8.63–24.21)	−73.7%
14 ± 2 months post‐op (FU)	1.14 ± 0.38 (0.85–1.43)	−83.4%	4.69 ± 4.27 (1.47–7.91)	−76.1%	91.30 ± 8.13 (85.48–97.12)	+76.2%	16.89 ± 11.54 (8.41–25.37)	−72.9%
*F*‐value	350.00	—	54.65	—	89.41	—	48.49	—
Overall *P*‐value	< 0.01	—	< 0.01	—	< 0.01	—	< 0.01	—
Paired *P*‐values	Pre vs. 1 week < 0.01	—	Pre vs. 1 week < 0.01	—	Pre vs. 1 week < 0.01	—	Pre vs. 1 week < 0.01	—
	Pre vs. FU < 0.01		Pre vs. FU < 0.01		Pre vs. FU < 0.01		Pre vs. FU < 0.01	
	1 week vs. FU < 0.01		1 week vs. FU > 0.05		1 week vs. FU > 0.05		1 week vs. FU > 0.05	

Abbreviation: FU, follow‐up.

### 3.3 Functional Pain Outcomes

**FIGURE 4:**
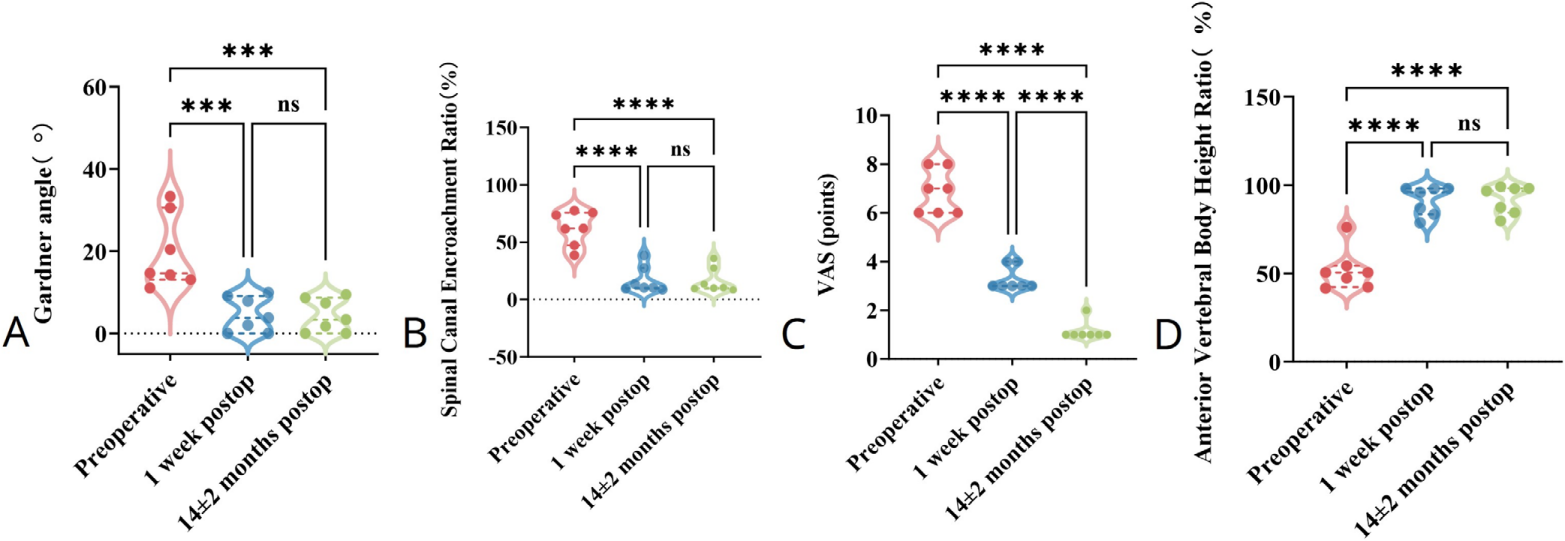
Violin plots illustrating changes in clinical and radiographic parameters at three time points: Preoperatively, 1 week postoperatively, and at 14 ± 2 months postoperatively. (A) VAS scores for low back pain significantly decreased postoperatively and continued improving at final follow‐up. (B) Gardner angle markedly decreased postoperatively and remained stable, indicating sustained correction of kyphotic deformity. (C) Anterior vertebral height ratio significantly improved postoperatively and was maintained throughout follow‐up. (D) Spinal canal encroachment ratio substantially decreased following decompression and remained stable at the 14 ± 2‐month follow‐up.

Low back pain, assessed by VAS scores, improved significantly over time (repeated‐measures ANOVA: *F* = 350.00, *p* < 0.001). Mean VAS scores decreased from preoperatively 6.86 ± 0.90 to 3.29 ± 0.49 at 1 week postoperatively and further to 1.14 ± 0.38 at 14 ± 2 months follow‐up (*p* < 0.01 vs. preoperative; *p* < 0.01 vs. 1 week postoperative), demonstrating an 83.4% reduction in pain intensity (Figure 4, Table 2).

### 3.4 Radiographic Assessment

All vertebral fractures healed successfully. The Gardner angle improved significantly from 19.63° ± 8.92° preoperatively to 4.39° ± 4.08° at 1 week postoperatively and remained stable at 4.69° ± 4.27° at the final follow‐up (*F* = 54.65, *p* < 0.01). Similarly, the anterior vertebral height ratio significantly improved from 51.82% ± 1.20% preoperatively to 91.96% ± 7.93% at 1 week postoperatively, remaining stable at 91.30% ± 8.13% at final follow‐up (*F* = 89.41, *p* < 0.01). No statistically significant differences were observed between postoperative and final follow‐up values (*p* > 0.05), indicating sustained vertebral height restoration and deformity correction (Figure 4B,C).

Postoperative CT revealed that the spinal canal encroachment ratio significantly decreased from 62.32% ± 14.90% preoperatively to 16.42% ± 10.78% at 1 week postoperatively and 16.89% ± 11.54% at final follow‐up. These changes were statistically significant compared to preoperative values (*F* = 48.49, *p* < 0.01), confirming effective spinal canal decompression. No statistically significant difference was identified between 1‐week postoperative and final follow‐up measurements (*p* > 0.05), indicating stable spinal canal morphology without recurrence or progression (Figure 4D).

## 4 Discussion

The surgical management of thoracolumbar burst fractures with neurological deficits requires a delicate balance between achieving complete neural decompression and preserving spinal stability. Traditional posterior approaches, while providing adequate exposure, often necessitate extensive disruption of the posterior musculoligamentous complex, potentially compromising long‐term functional outcomes [3, 4, 17]. This clinical dilemma has driven the exploration of muscle‐sparing techniques that can address both objectives simultaneously. This study demonstrated that a modified posterior hybrid approach combining the Wiltse interval and SPSA is technically feasible and achieves satisfactory early clinical and radiographic outcomes in thoracolumbar burst fractures with neurological deficits. All seven patients achieved successful fracture reduction, restoration of anterior vertebral height, and bony union of the split spinous process. Neurological function improved by at least one ASIA grade in every case, and the mean VAS score for low back pain decreased markedly from 6.86 preoperatively to 1.14 at final follow‐up, representing an 83% reduction. The mean operative time was 172 min, with moderate intraoperative blood loss and no major complications.

### 4.1 Rationale for Technique Development: Limitations of Conventional Approaches

The development of this modified hybrid technique was motivated by the recognized limitations of conventional surgical approaches for thoracolumbar burst fractures with neurological deficits. For thoracolumbar fractures without neurological deficits, posterior fixation via the Wiltse paraspinal intermuscular approach alone is often sufficient to achieve stabilization while minimizing muscle trauma [18]. However, in the presence of neurological impairment, decompression becomes mandatory in addition to fracture stabilization [19].

The conventional open posterior approach remains widely used because it offers broad surgical exposure and facilitates direct decompression and fixation. Nevertheless, this approach requires wide subperiosteal muscle stripping and prolonged retraction, often involving removal of the spinous processes and their ligamentous attachments. These maneuvers may lead to muscle ischemia, fatty infiltration, secondary fibrosis, and potential postoperative instability [20, 21, 22, 23, 24, 25, 26]. Such structural alterations have been closely linked to chronic postoperative low back pain. In the present study, patients demonstrated a substantial reduction in VAS scores (from 6.86 to 1.14, representing an 83% decrease), which may, at least in part, be attributable to the preservation of posterior soft tissues achieved by the modified technique.

Although combined anterior–posterior approaches can achieve effective neural decompression and robust reconstruction, they generally require two surgical stages, increasing operative complexity and duration. Previous reports have documented longer hospitalization and higher complication rates in patients undergoing combined procedures [13]. Compared with these findings, the mean operative time of 172 min and absence of major complications observed in our study suggest that a muscle‐preserving posterior hybrid strategy may represent a less invasive alternative while maintaining adequate decompression and stabilization.

### 4.2 Biomechanical Rationale and Structural Preservation

Since its initial application for lumbar spinal stenosis in 2005, SPSA has become widely adopted in posterior lumbar decompression and fusion procedures [27]. However, studies reporting the application of SPSA specifically for thoracolumbar burst fractures with neurological deficits remain scarce, particularly with respect to radiological maintenance of alignment and clinical outcomes. In the present study, significant improvements were observed in Gardner angle, anterior vertebral height ratio, and spinal canal encroachment ratio after surgery, and these corrections were maintained at final follow‐up without progressive kyphotic deformity or fixation failure. These findings suggest that SPSA can provide adequate decompression while preserving postoperative segmental stability in thoracolumbar burst fractures.

The structural characteristics of SPSA may explain these favorable outcomes. This technique involves a midline longitudinal split of the spinous process, followed by detachment at its base while preserving lateral muscular attachments. In contrast, conventional posterior midline approaches typically require bilateral subperiosteal elevation of paraspinal muscles and removal of the spinous processes together with the supraspinous and interspinous ligaments. Once disrupted, this posterior osteoligamentous complex cannot be anatomically restored and may predispose patients to decreased spinal stability [11]. Such structural disruption has been associated with altered segmental biomechanics and an increased risk of adjacent segment degeneration following interbody fusion procedures [28].

Compared with previously reported conventional posterior approaches for thoracolumbar burst fractures, where varying degrees of correction loss during follow‐up have been described, the absence of significant alignment deterioration in our cohort (Gardner angle: 4.39° at 1 week vs. 4.69° at final follow‐up; anterior vertebral height ratio: 91.96% vs. 91.30%) may be related to preservation of the posterior tension band. Although direct biomechanical testing was not performed, our radiological findings provide indirect clinical support for the theoretical structural advantages of SPSA.

### 4.3 Comparison of Clinical Outcomes With Previous Studies

The clinical outcomes observed in our cohort are generally consistent with previous studies evaluating SPSA in lumbar decompression and fusion procedures. Previous comparative studies have demonstrated that SPSA results in lower postoperative serum creatine kinase levels and reduced multifidus muscle atrophy on MRI compared with conventional open posterior surgery, particularly at fused segments and adjacent caudal levels, as summarized in Table 3. Furthermore, lower VAS scores for low back pain at 1 and 3 years postoperatively have been reported, and the extent of multifidus atrophy was positively correlated with residual discomfort, highlighting the long‐term symptomatic benefit of muscle preservation [11, 12, 30, 38, 42, 43].

**TABLE 3:** Literature review of SPSA.

Number	Study	Method	Summary of clinical outcomes	Imaging results	Conclusion
1	Kurogochi et al. [29]	SPSL vs. PLF	JOA/ODI/VAS: no significant change; slippage progression rate: no significant change	PT decreased (SPSL); LL increased (PLF)	SPSL: stability increased; decompression increased; no instrumentation and fusion
2	Liu et al. [30]	MHSPS‐TLIF	JOA increased; VAS decreased	Visualization increased; operating time decreased; muscle injury decreased	Decompression increased; atrophy decreased
3	Voglis et al. [31]	SPSL vs. CD	Pain decreased (POD3/5); functional recovery: no significant difference	Decompression increased; atrophy decreased	SPSL: pain decreased; analgesic use decreased
4	Ovalioglu et al. [32]	SPSD vs. CL	ODI decreased; VAS decreased	APD increased by 72.2%; CSA increased by 102.5% (comparable to CL)	SPSD: muscle‐sparing; decompression similar to CL
5	Masuda et al. [33]	Mod. Marmot vs. LSPSL	JOA increased; recovery rate increased; ODI: no significant difference	Early pain decreased (Mod. Marmot); long‐term outcomes similar (LSPSL)	LSPSL: long‐term recovery increased
6	Okubo et al. [34]	SPSL‐CM/CE	JOA increased; JOA‐BPEQ increased	TK: no change; LL: no change (2 years)	Stability increased; tumor resection feasible
7	Tomasi et al. [35]	LSPST	Novel atrophy classification; volume analysis	Cyst resection complete; muscle volume unchanged	MIS cyst/LSS resection; muscle preservation increased
8	Tanaka et al. [36]	LSPSL vs. TISLD	JOA/JOA‐BPEQ: no significant difference; disc height loss: TISLD > LSPSL	L4/5 disc height decreased (TISLD); minimal disc height loss (LSPSL)	LSPSL: disc height increased; stability increased
9	Tarabay et al. [37]	SPSL‐NTLEH	Muscle power increased; neurological recovery increased; motor function increased (6 months)	Decompression complete; compression decreased	Pediatric‐friendly; avoids growth disturbance and atrophy
10	Liu et al. [38]	SPS‐TLIF	Operation time decreased; blood loss decreased; low back pain decreased; atrophy at 2 years decreased vs. conventional	Paraspinal muscle preservation increased; atrophy decreased	Minimally invasive; complications decreased; recovery increased
11	Oyama et al. [39]	MEL vs. SPSL	Operation time increased; length of stay decreased; pain: no significant difference; JOA‐BPEQ: no significant difference	Imaging: no significant difference	SPSL: efficiency increased; MEL: recovery increased
12	Chen et al. [40]	LSPSL vs. CL	Efficacy: no significant difference; complications: no significant difference; atrophy decreased (LSPSL)	Operation time: no significant difference; blood loss: no significant difference; imaging: no significant difference	LSPSL: atrophy decreased (significant)
13	Kato et al. [41]	SPAS–OLF	Recovery rate increased; muscle injury decreased; return to work ≤ 3 months; no deformity or recurrence	Decompression increased; muscle injury none	Suitable for athletes and high‐demand patients

Abbreviations: APD, anteroposterior diameter; BL, blood loss; CL, conventional laminectomy; CSA, cross‐sectional area; DH, disc height; IF, instrumentation and fusion; JOA, Japanese Orthopedic Association (score); JOA‐BPEQ, Japanese Orthopedic Association Back Pain Evaluation Questionnaire; LBP, low back pain; LL, lumbar lordosis; LOS, length of stay; LSPSL, lumbar spinous process–splitting laminectomy; LSS, lumbar spinal stenosis; MEL, microendoscopic laminotomy; MI, muscle injury; MIS, minimally invasive surgery; MP, muscle power; ODI, Oswestry Disability Index; OLF, ossification of the ligamentum flavum; OT, operating time; PLF, posterolateral fusion; PT, pelvic tilt; RR, recovery rate; SPSA, spinous process–splitting approach; SPSD, spinous process–splitting decompression; SPSL, spinous process–splitting laminectomy; TK, thoracic kyphosis; TLIF, transforaminal lumbar interbody fusion; VAS, visual analogue scale.

Consistent with these findings, we observed a marked reduction in low back pain in the present study. The mean VAS score decreased from 6.86 preoperatively to 1.14 at final follow‐up, representing an 83% reduction. Although serum biomarkers and quantitative MRI muscle assessments were not performed, this degree of pain improvement may indirectly reflect the reduced posterior soft‐tissue injury achieved through the muscle‐preserving characteristics of SPSA.

Regarding neurological outcomes, all patients in our series improved by at least one ASIA grade, with five patients improving from grade C to D and two from grade D to E. Although direct comparisons with previous cohorts are limited by differences in injury severity and classification systems, these results are comparable to those reported in earlier studies of posterior decompression for thoracolumbar burst fractures [19, 21]. The consistent neurological improvement observed in our cohort further supports the adequacy of neural decompression achieved with this modified surgical approach.

### 4.4 Perioperative Outcomes and Complications

Clinically, randomized controlled and comparative studies have indicated that SPSA provides superior acute and subacute pain control without increasing analgesic requirements, thereby facilitating earlier ambulation and postoperative rehabilitation [44]. Although we did not perform a direct comparative analysis, the moderate operative time, acceptable intraoperative blood loss, absence of major complications, and stable postoperative alignment observed in our cohort suggest that the modified hybrid technique maintains favorable perioperative safety while extending the application of SPSA to more complex fracture cases.

Long‐term follow‐up studies beyond 5 years have demonstrated that SPSA reliably maintains segmental stability and sagittal alignment, with low reoperation rates and frequent osseous union of the split spinous process [45]. In our series, radiographic union was achieved in all cases without loss of correction, and no kyphotic progression was detected during follow‐up. Although our follow‐up duration was shorter, these findings remain consistent with previously reported long‐term stability outcomes.

Regarding complications, four patients (57.1%) in our cohort were found to have pre‐existing dural tears caused by fracture fragments, all of which were recognized and repaired intraoperatively. This relatively high incidence likely reflects the severity of canal compromise in our selected population (mean preoperative spinal canal encroachment ratio: 62.32%) rather than a limitation of the surgical technique. The single case of postoperative cerebrospinal fluid leakage resolved with conservative management, suggesting that the SPSA approach provides adequate surgical visualization for meticulous dural repair.

### 4.5 Limitations and Future Perspectives

This study has several limitations that warrant consideration. First, the single‐center retrospective design with a small sample size consisting exclusively of male patients substantially limits the generalizability of the results. The absence of female participants may influence the applicability of our findings, as sex‐related differences in bone quality, spinal biomechanics, hormonal influences, and postoperative recovery could potentially affect surgical outcomes. Future studies including female patients are required to validate the broader applicability of this modified approach. Selection bias cannot be excluded, as the surgical approach was determined by surgeon preference rather than randomization. Second, the small sample size (*n* = 7) inherently limits the statistical power of repeated‐measures ANOVA, and therefore, all statistical findings should be interpreted with caution. Third, the absence of a control group treated with conventional posterior or combined anterior–posterior approaches precludes direct comparison and definitive conclusions regarding the comparative efficacy of this approach. Fourth, the relatively short follow‐up duration may not capture long‐term outcomes, such as adjacent segment degeneration, implant failure, or delayed neurological deterioration. In addition, two key issues warrant further investigation: (1) the biomechanical advantages of preserving the spinous process and paraspinal tension band have not yet been quantitatively validated through finite element modeling or experimental studies; and (2) although this technique demonstrates good technical feasibility, its learning curve, reproducibility, and cost‐effectiveness in broader clinical practice remain to be evaluated. Future multicenter, prospective studies incorporating larger, gender‐diverse cohorts, standardized follow‐up and control groups are needed to confirm the long‐term safety, efficacy, and optimal indications of this combined approach.

## 5 Conclusions

The modified SPSA combining the Wiltse interval and midline spinous process splitting appears to be a feasible option for neural decompression, anatomic reduction, and fixation in selected patients with thoracolumbar burst fractures and neurological deficits, while maximally preserving the posterior osteoligamentous complex and minimizing paraspinal muscle trauma. In this preliminary series of seven patients, ASIA grades improved by at least one level, and mean low back pain VAS scores decreased from 6.86 preoperatively to 1.14 at 14 ± 2 months postoperatively. Radiographic parameters, including the Gardner angle, anterior vertebral height ratio, and spinal canal encroachment ratio, improved significantly and remained stable during follow‐up. All fractures achieved bony union without loss of correction or progression of kyphosis. Given the retrospective design, limited sample size, and absence of a control group, larger prospective comparative studies are necessary to validate the efficacy and safety of this approach and define its indications. Future multicenter randomized controlled studies are warranted to evaluate whether this technique offers advantages over conventional methods in terms of neurological recovery, deformity correction, and soft tissue preservation.

## Author Contributions

All authors contributed to the study conception and design. The first draft of the manuscript was written by Kaixuan Chen. Yizhong Ma and Zihui Yang performed the figure preparation and statistical analyses. Hongfeng Ruan and Guanyi Liu supervised the study and critically revised the manuscript. All authors have read and approved the final manuscript.

## Funding

This study was financially supported by the Ningbo Public Welfare Research Plan Key Project (No. 2023S036) and Ningbo Clinical Research Center for Orthopedics, Sports Medicine & Rehabilitation (2024L004).

## Ethics Statement

The study was approved by the Ethics Committee of Ningbo No. 6 Hospital, Ningbo, China (approval no. 202335K).

## Consent

Written informed consent was obtained from all participants before enrollment in the study.

## Conflicts of Interest

The authors declare no conflicts of interest.

## Data Availability

The data that support the findings of this study are available on request from the corresponding author. The data are not publicly available due to privacy or ethical restrictions.
